# Developing critical thinking and decision-making skills for cancer information: the Informed Health Choice-Cancer online learning resource

**DOI:** 10.1007/s11764-025-01874-6

**Published:** 2025-08-07

**Authors:** Mengqi Li, Declan Devane, Claire Beecher, Maura Dowling, Austin G. Duffy, Caitriona Duggan, David Robert Grimes, Avril Kennan, Claire Kilty, Allen Nsangi, Andrew D. Oxman, Derek C. Stewart, Elaine Toomey, Marie Tierney

**Affiliations:** 1 School of Nursing & Midwifery, University of Galway, Galway, Ireland; 2 Health Research Board – Trials Methodology Research Network (HRB-TMRN), University of Galway, Galway, Ireland; 3 Evidence Synthesis Ireland and Cochrane Ireland, University of Galway, Galway, Ireland; 4 Department of Medical Oncology, Mater Misericordiae University Hospital, Dublin, Ireland; 5 Department of Oncology, Portiuncula University Hospital, Galway, Ireland; 6 Biostatistics Unit, Discipline of Public Health and Primary Care, School of Medicine, Trinity College Dublin, Dublin, Ireland; 7 Health Research Charities Ireland (HRCI), Dublin, Ireland; 8 Irish Cancer Society, Dublin, Ireland; 9 Department of Medicine, College of Health Sciences, Makerere University, Kampala, Uganda; 10 Centre for Epidemic Interventions Research, Norwegian Institute of Public Health, Oslo, Norway; 11 College of Medicine, Nursing & Health Sciences, University of Galway, Galway, Ireland

**Keywords:** Online cancer education, Critical thinking, Informed decision-making, Learning resource development, Misinformation, Patient and public involvement

## Abstract

**Purpose:**

People impacted by cancer face increasing challenges in navigating a landscape of abundant health information and misinformation. This study outlines the development and pilot testing of the Informed Health Choices-Cancer (IHC-C) learning resource, designed to provide users the skills and knowledge necessary to think critically about the reliability of health claims and make well-informed choices. This study outlines the development and pilot testing of this learning resource.

**Methods:**

Using a human-centred design approach, the resource was co-developed with those impacted by cancer through iterative refinement. Tailored content and multimedia formats were used to support over three stages of pilot testing: initial prototype development, testing with small stakeholder groups, and testing with a broader sample. Quantitative ratings and qualitative feedback guided improvements in accessibility, usability, and relevance.

**Results:**

Thirty-three participants responded positively to the resource, noting its accessible language, practical content, and engaging format. In the final round of testing, over 80% found the resource as ‘very well aligned’ with learning outcomes, ‘very easy to understand’, ‘very relevant’ to cancer, and ‘very easy to navigate’. Navigation ratings improved from 50.0% at prototype stage to 85.4% at final stage. The resource was perceived as effective in supporting critical thinking, informed decision-making, and reflecting real-life cancer experiences.

**Conclusions:**

The IHC-C learning resource offers an innovative, practical tool for integrating critical thinking into cancer care. It shows promise in supporting informed decision-making and reduces vulnerability to misinformation.

**Implications for Cancer Survivors:**

The resource may enhance health literacy and empower survivors to critically evaluate health information and manage their care more confidently.

## Introduction

The International Agency for Research on Cancer (IARC) reported nearly 20 million new cancer cases and 9.7 million cancer-related deaths in 2022, underscoring a significant global health burden [1]. Projections indicate that new cancer cases may reach 35 million by 2050 [1]. This increasing prevalence underscores the urgent need for robust, accessible health information to support those impacted — patients, survivors, caregivers, and loved ones alike [2] by cancer in making critical decisions from diagnosis throughout their cancer journey, which requires precise and actionable information [3–7].

However, while cancer information is now more readily available, with approximately one in three patients seeking information independently [8], many still struggle to meet their information needs [6, 7]. For example, 91.9% of breast cancer patients report obtaining cancer-related information from television, friends, and family, while over half use the internet or healthcare providers [9, 10]. Despite this abundance of information, there are challenges in identifying and navigating reliable sources [11]. Studies highlight significant variability in the quality of cancer-related information, making it one of the most frequently raised concerns regarding health information [11–13], often leading to confusion.

Social media further complicates this landscape by often disseminating low-quality or commercially biased health information across many cancer types and platforms without adequate vetting [14, 15]. This has contributed to an ‘infodemic’ of misinformation, a term coined by the World Health Organization. Misinformation refers to inaccurate, outdated, incomplete, or misleading information disseminated without necessarily intending to deceive [16–18].

Few areas of health have been as insidiously impacted by misinformation as cancer, which not only skews public perception but also influences patients’ decisions and behaviours [2]. Analysis of the 200 most popular social media articles about the four most common cancers revealed that 32.5% contained misinformation and 30.5% contained harmful information. Of the articles containing misinformation, 76.9% also contained harmful information [19]. For prostate cancer specifically, studies found that 41% of the 55 top TikTok posts and 40% of 126 Instagram posts contained misinformation [20, 21]. A study of 540 Pinterest posts on various cancers revealed misinformation rates ranging from 4 to 26% [22], and 44% of evaluated cancer-related tweets contained misinformation, with 31% containing harmful information and 30% including both [23]. It has also been found that misleading social media content is significantly more engaging, with more views and likes [23–26], and 28 times more likely to be shared [27]. Over 70% of people sometimes accept such misinformation as truth [16], with cancer patients being particularly vulnerable [28], as health literacy among those impacted by cancer, including individuals with a diagnosis and caregivers, is reported to be at a low to moderate level [29–31]. As a result, those misinformed may delay effective treatments, choose unproven alternatives, and ultimately face poor health outcomes and decisional regrets [16, 32].

A range of interventions and strategies have been introduced in efforts to address misinformation. These include engaging healthcare professionals in social networks to foster comprehensive, evidence-based dialogues and content across the cancer care ecosystem [33–35], developing recommendations for oncologic advice providers [28], and identifying strategies with cancer clinicians [36]. While these approaches are practical, they are limited by the vast scale of misinformation and the intensive effort required to counteract it continually.

A promising approach has been enhancing health literacy through education in easy-to-understand languages [35, 37–40]. This involves improving skills and knowledge through clear, plain-language education. Enhanced health literacy enables people to better understand, evaluate, and make informed choices about claims and comparisons, reducing the number and extent to which they are potential victims of false and misleading information [17, 18, 41]. While improving health literacy is essential, it alone may not equip individuals to critically navigate misinformation. Strengthening critical thinking and decision-making skills builds on the foundation of health literacy by enabling people to question, evaluate, and apply information more effectively in real-world contexts.

Recognising the need for resources that explicitly support both health literacy and critical thinking, the Informed Health Choices (IHC) initiative, led by an international collaboration of researchers, employs a structured, evidence-based approach to create and evaluate educational resources that enable people of all ages to recognise reliable claims and make informed health choices critically [42]. The initiative includes 49 Key Concepts (KCs) that help people recognise unreliable claims, identify reliable evidence, and make well-informed choices [43]. These KCs have been successfully adapted across various demographics and disciplines, significantly enhancing critical thinking and decision-making skills among primary and secondary school children [44–47], parents [38], healthcare students [48], and the broader population [49].

Building on this foundation, we have developed the Informed Health Choices-Cancer (IHC-C) programme to provide those impacted by cancer with the skills and knowledge necessary to think critically about the reliability of health claims and make well-informed choices. The IHC-C is structured in two stages. The first stage involves prioritising the most relevant KCs for inclusion in this programme [2, 50]; the second stage consists of developing an evidence-based online learning resource based on the prioritised KCs, which will be ready for a randomised trial. This paper details the development process and the anticipated impact of the online learning resource.

## Methods

### Design

**Fig. 1:**
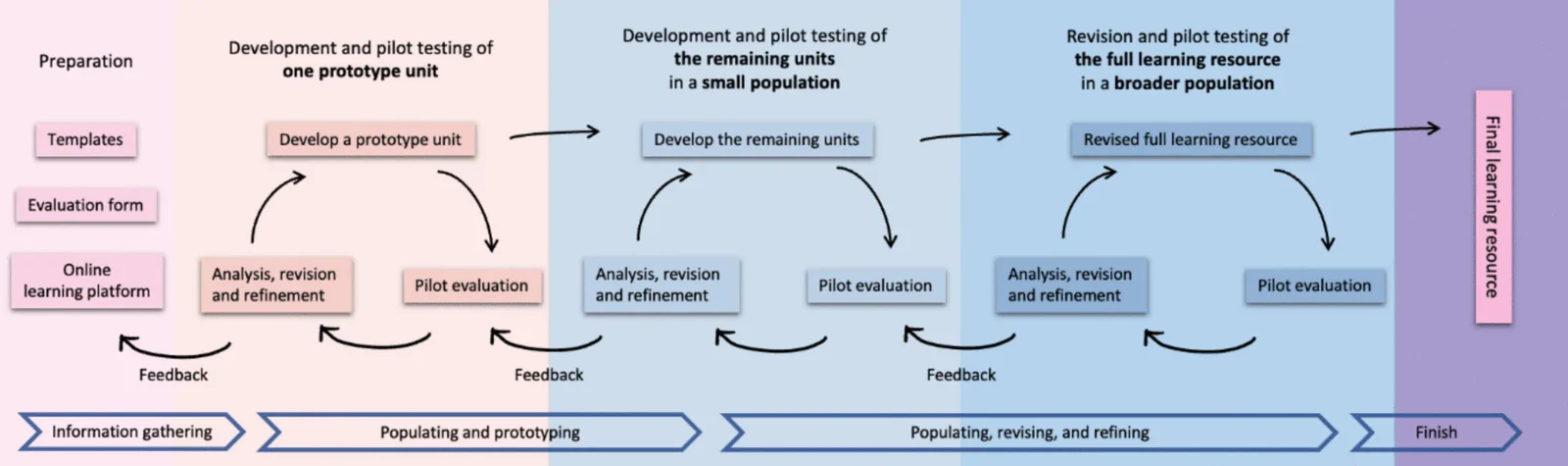
Iterative development and pilot testing process

A human-centred design (HCD) approach with iterative refinement cycles was used to develop the learning resource over 17 months (from May 2023 to October 2024) [51, 52]. This process involved prototyping, collecting feedback, and making iterative adjustments. Initial feedback and revisions during the development and pilot testing of a prototype unit laid the groundwork for the development, refining, and testing of the remaining units (see Fig. 1). Key stakeholders, including those impacted by cancer, healthcare professionals, and researchers, were selected purposively based on their expertise or lived experiences [2]. They actively contributed throughout the development and piloting by providing iterative feedback via pilot testing sessions, ensuring a robust and inclusive refinement process that reflected diverse perspectives. Ethics approval was granted by the University of Galway Research Ethics Committee (reference 2022.03.005).

### Materials

The learning resource comprises nine units, each based on one of the KCs prioritised during the initial stage of the IHC-C programme [50]. These KCs were selected for their distinct roles in fostering critical thinking and guiding decision-making in cancer-related contexts. Following input from the stakeholders, the titles of these ‘Key Concepts’ were revised to ‘Guiding Principles’ to enhance readability and clarity for the general public during both the prioritisation and development stages (see Table 1) [50].

**Table 1:** Comparisons of the revisions of prioritised units’ titles

No	Original Key Concepts’ titles	Revised Key Concepts’ titles	Guiding Principles’ titles
1	Do not assume that treatments are safe	Not many health treatments that work are 100% safe	Not all medicines or treatments that help us get better are completely safe
2	Do not assume that treatments effects are certain	We can rarely be 100% sure that a treatment works or is safe	It is often difficult to be completely certain that a treatment works or is safe
3	Do not assume that association is the same as causation	Just because a health treatment is linked with people getting better or worse, that doesn’t mean it was the cause of people getting better or worse	Just because a health treatment is linked to people improving or getting worse doesn't automatically mean it's the reason for these changes
4	Do not assume that a single study is sufficient	The results of single studies can be misleading	The results of single studies can be misleading
5	Do not assume that a treatment is better based on how new or technologically impressive it is	Not all ‘new’ treatments are better	New or expensive treatments are not always better
6	Do not assume that your beliefs are correct	Don’t let what you believe lead you down the wrong path about a health treatment	Question beliefs to make better health decisions
7	Consider whether the people being compared were similar	Consider whether the people being compared in a study were similar	Consider whether the people being compared in a study were similar
8	Weigh the benefits and savings against the harms and costs of acting or not	Does a health treatment have more advantages than disadvantages for you?	Evaluating the facts: Balancing advantages and disadvantages in health choices
9	Consider how important each advantage and disadvantage is when weighing the pros and cons	When you are making a choice about a health treatment, think about how important each advantage and disadvantage is	Personal considerations: Prioritising what matters most in your personal health choices

### Develop templates

**Fig. 2:**
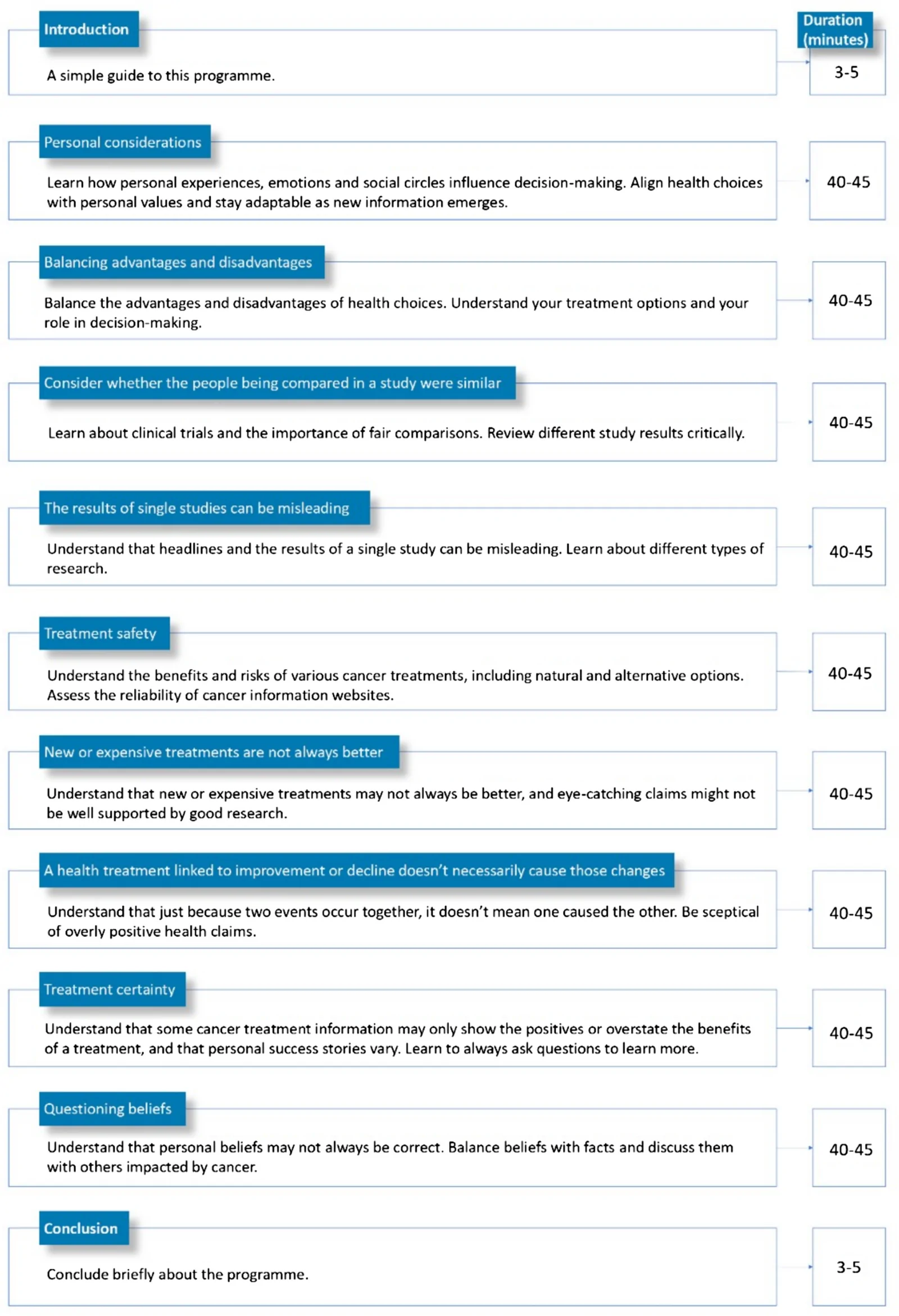
Structure of the learning resource

A ‘learning resource template’ was created to structure the unit flow, define and align the units’ aims with the learning outcomes, and specify the duration (see Fig. 2).

**Fig. 3:**
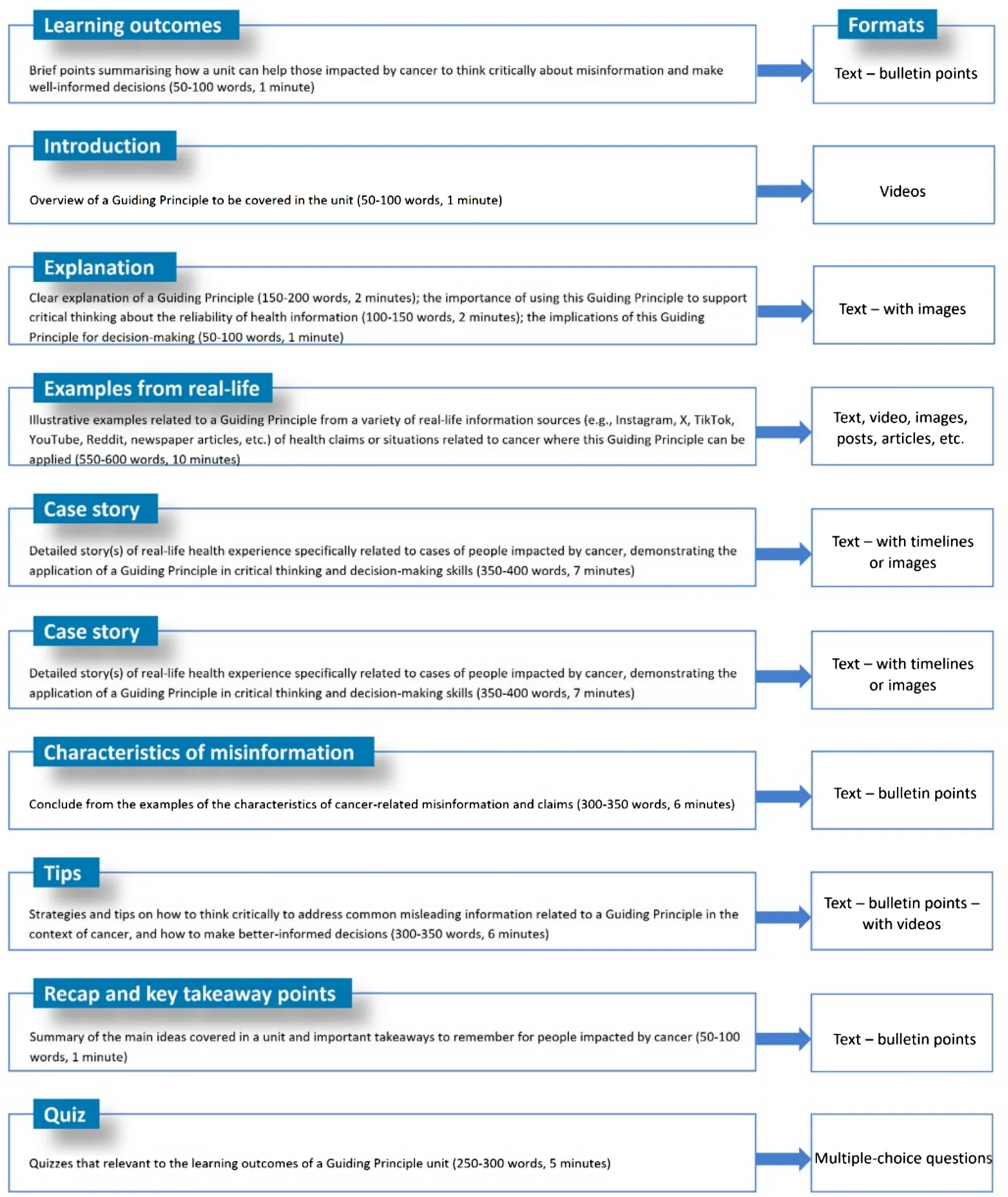
Unit template

A ‘unit template’ was also created to structure each unit, outlining the learning outcomes, sections, indicative content, and core resources (see Fig. 3). The template specified that each unit should include various educational formats to provide information, including narrated texts, videos, online articles, social media posts, and interactive activities, ensuring comprehensive and effective dissemination of cancer-specific information and knowledge.

### Identify an online learning management platform

We compared open-source learning management system (LMS) platform options against specific criteria relevant to cancer education delivery (e.g. flexibility and customisation capabilities, comprehensive features, user-friendliness, cross-platform accessibility, and cost-effectiveness) [53, 54]. Moodle, an open-source learning platform widely used in education, best aligned with these requirements and was selected as the final platform.

### Develop and pilot test one prototype unit

#### Populate the prototype unit

The prototype unit was developed using the established unit template based on a prioritised KC. All sections of the template were populated with relevant, cancer-specific content drawn from a wide range of source materials to emphasise aspects unique to the KC topic, ensuring precise alignment with the targeted learning outcomes, and providing a comprehensive and contextual understanding of the KC (see Fig. 3). These source materials included:

- National and international health guidelines (e.g. WHO, NICE)
- Peer-reviewed research evidence (e.g. PubMed)
- Popular social media platforms featuring widely shared real-world examples (e.g. Facebook, Instagram, TikTok)
- News outlets and aggregator platforms that appear prominently in search results (e.g. Google News, The Independent)

To promote transparency and help users understand where information came from, each content item was tagged with a source label indicating its origin (e.g. TikTok post, published 14 June 2024). In addition, a clear, user-facing note was presented at the beginning of the learning resource stating that some examples in the learning resource might include misleading information; that certain words in these examples might seem similar or interchangeable but can actually mean different things; and that the content is for learning only and does not constitute medical advice.

The development process involved collaborative work among researchers, including an iterative process of drafting, reviewing, revising, and refining, aimed at improving each unit to meet high educational standards and effectively communicate the intended learning outcomes. The prototype unit was then piloted into the selected online learning platform.

#### Agree on an evaluation form

A comprehensive, user-friendly evaluation form was developed to effectively evaluate user experiences at the end of each unit. The form integrates open-ended and rating-scale items, with questions focusing on alignment with intended learning outcomes, ease of understanding, relevance to those impacted by cancer, and ease of navigation. These outcomes were selected by drawing on experience from existing resources in the field to ensure the content addressed user needs and was feasible to measure. Participants rated each question using a 4-point scale (e.g. ‘not at all’, ‘not very’, ‘somewhat’, ‘completely’) to indicate the extent to which each criterion was met. Open-ended questions invited participants to share what they found effective and to suggest improvements.

#### Pilot testing of the prototype unit

##### Participant recruitment

Participants involved in the development and pilot testing phases were recruited primarily from two existing groups established during the earlier prioritisation stage of the IHC-C programme: the project group and the patients and carer participation group, as detailed elsewhere [47]. Additional participants were recruited through expressions of interest received during earlier outreach efforts and through targeted promotion on social media platforms. Purposive sampling was used to ensure diversity in cancer experience, age, gender, and educational background. All participants received study information and gave informed consent prior to participation.

##### Participants

Participants from both groups were involved throughout the development and pilot testing phases. The purposive sample approach included individuals with a range of key demographics, including cancer experience (e.g. patients, survivors, caregivers and loved ones), and reflected diversity in gender, age, and educational background.

##### Process

Participants who accepted the invitation were enrolled in the study. Instructions were provided for accessing the learning resource and using the evaluation form. A review period of 2 weeks was allocated to allow sufficient time for thorough engagement with and evaluation of the prototype unit. Email assistance was available to support learners, with responses typically provided within 24 h.

##### Feedback analysis and revision

Quantitative feedback data was summarised descriptively using SPSS (version 28.0), and valid percentages were calculated where necessary. Qualitative feedback data was analysed by identifying keywords and phrases and clustering associated terms into themes using NVivo (version 20 Pro) to guide further improvement. Based on this analysis, modifications were made to both the prototype unit and the unit template to ensure consistency with the revised unit format. Rationales were provided for any feedback not incorporated into the revisions.

By the conclusion of this pilot, both a refined prototype unit and an updated unit template had been established, and they were ready for further development of the remaining units.

### Develop and pilot test the full learning resource

#### Populate the remaining units

Following the refined unit template, all remaining units were populated and tailored using similar procedures outlined in the ‘Develop the prototype unit’ section. Subsequently, each unit was integrated into the selected online platform for pilot testing.

#### Pilot testing the full learning resource

The pilot testing was conducted in two sequential cycles (see Fig. 1): an initial cycle with a small participant group to identify and address major issues and a broader testing cycle. This staged approach enabled thorough refinement of the learning resource before broader implementation.

#### Cycle 1: with a small group of participants

##### Participants

Purposive sampling was used to ensure participant diversity (e.g. age group, cancer experience, and educational background). Participants in the patients and carer participation group who had been involved in this IHC-C programme since the prioritisation stage were invited [50].

##### Process, feedback analysis, and revision

This pilot process and feedback analysis followed the methods used for the prototype unit, with an additional evaluation question added to rank units and improve resource flow [2, 50]. Furthermore, a cross-unit linkage method was applied to link the feedback from each unit, enhancing the coherence of the refinement of the learning resource. A 3-week review period was allocated for this pilot. Adjustments and revisions were made to each unit to ensure they met the needs and expectations of the target audience.

#### Cycle 2: with a broader group of participants

##### Participants

A diverse group of participants with varied cancer experience, age ranges, and educational backgrounds was invited for this pilot cycle, including:

- Participants in the patients and carer participation group who completed the prioritisation stage,
- Potential patients and carers who expressed interest during the prioritisation stage recruitment of this IHC-C programme but were unable to attend,
- New recruits were sourced through social media platforms.

##### Process, feedback analysis, and revision

This process and feedback analysis mirrored that of cycle 1. Necessary adjustments and revisions were made to refine each unit again. The units were then updated on the online learning platform for pilot testing. A 3-week review period was allocated for comprehensive evaluation and refinement.

By the end of this pilot, a robust learning resource had been established, ready for effectiveness testing in a randomised trial.

## Results

### Development and pilot testing of the prototype unit

#### Participants

A total of 15 people who had attended the prioritisation were invited to participate in the prototype unit’s pilot testing. All (100.0%) agreed to participate and 14 participants (93.3%) completed the process (see Table 2). Ten of these participants were members of the IHC-C programme’s project group [50].

**Table 2:** Sociodemographic characteristics of participants

	**Prototype unit**	**Full learning resource pilot testing — cycle 1**	**Full learning resource pilot testing — cycle 2**
	*n* = 14 (%)	*n* = 3 (%)	*n* = 16 (%)
Gender			
Female	8 (57.1)	1 (33.3)	14 (87.5)
Male	6 (42.9)	2 (66.7)	2 (12.5)
Age			
18–24	0	1 (33.3)	0
25–34	0	0	1 (6.3)
35–44	6 (42.9)	1 (33.3)	4 (25.0)
45–54	3 (21.4)	0	5 (31.2)
55–64	2 (14.3)	1 (33.3)	2 (12.5)
Above 64	3 (21.4)	0	4 (25.0)
Ethnicity			
White	13 (92.9)	3 (100.0)	14 (87.5)
Black	1 (7.1)	0	1 (6.3)
Mixed group	0	0	1 (6.3)
Education			
Secondary education	0	0	4 (25.0)
Third level (primary degree)	1 (7.1)	3 (100.0)	3 (18.8)
Third level (postgraduate award)	13 (92.9)	0	9 (56.2)
How impacted by cancer			
Current cancer patients	2 (50.0)	0	3 (18.8)
Cancer survivors	1 (25.0)	1 (33.3)	7 (43.7)
Informal caregivers	1 (25.0)	1 (33.3)	3 (18.8)
Loved ones	0	1 (33.3)	3 (18.8)
Time since cancer diagnosis			
Median (range), years	2.2 (1.1, 2.8)	4.4 (3.3, 4.8)	5.9 (3.4, 12.0)

### Participants’ feedback

**Fig. 4:**
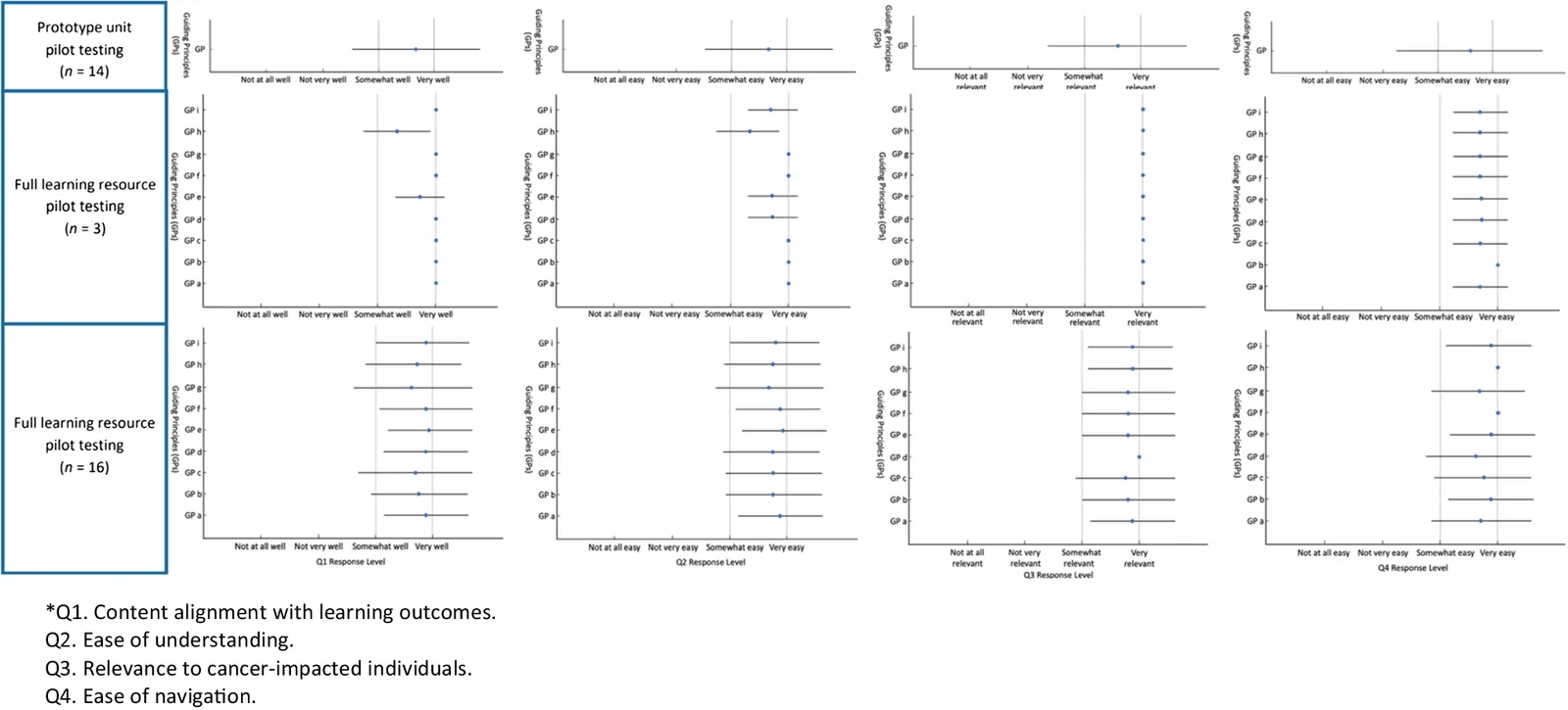
Evaluation questions results for pilot testing

Participant responses to the prototype unit evaluation (see Table 3 and Fig. 4) showed:

- **Content alignment with learning outcomes:** All participants rated the unit’s content as ‘well aligned’ with the learning outcomes, with 71.4% rating it ‘very well aligned’. Specifically, ratings for ‘very well aligned’ were 70.0% in the project group and 75.0% in the patient and carer participation group.
- **Ease of understanding:** All participants found the content ‘easy to understand’, with 71.4% rating it ‘very easy to understand’. This rating was 70.0% in the project group and 75.0% in the patient and carer participation group.
- **Relevance to cancer-impacted individuals:** All participants deemed the content of the unit ‘relevant’ to the needs of cancer-impacted individuals, with 64.3% rating it ‘very relevant’. The project group rated it at 50.0%, while the patient and carer participation group rated it at 100.0%.
- **Ease of navigation:** Overall, 92.9% of participants rated the unit ‘easy to navigate’, with 64.3% rating it ‘very easy to navigate’. This rate was 70.0% in the project group and 50.0% in the patient and carer participation group.

**Table 3:** Summary of participant ratings on evaluation questions

**Evaluation questions**	**Response level**	** Prototype unit**			** Full learning resource**			
		**Overall** **(*n* = 14)**	**Project group** **(*n* = 10)**	**PCP group(n = 4)**	**Cycle 1** **(*n* = 3)**		**Cycle 2** **(*n* = 16)**	
		Mean % of responses	Mean % of responses	Mean % of responses	Mean % of responses	Range across units (%)	Mean % of responses	Range across units (%)
Content alignment with learning outcomes	Very well	71.4%	70.0%	75.0%	88.9%	33.3–100.0	82.6%	62.5–93.8
	Somewhat well	28.6%	30.0%	25.0%	11.1%	33.3–66.7	16.0%	6.2–37.5
	Not very well	0	0	0	0	0	1.4%	6.2
Ease of understanding	Very easy	71.4%	70.0%	75.0%	77.8%	33.3–100.0	80.6%	68.8–93.8
	Somewhat easy	28.6%	30.0%	25.0%	22.2%	33.3–66.7	19.4%	6.2–31.2
Relevance to cancer-impacted individuals	Very relevant	64.3%	50.0%	100.0%	96.3%	66.7–100.0	84.7%	75.0–100.0
	Somewhat relevant	35.7%	50.0%	0	3.7%	33.3	15.3%	12.5–25.0
Ease of navigation	Very easy	64.3%	70.0%	50.0%	70.4%	66.7–100.0	85.4%	68.8–100.0
	Somewhat easy	28.6%	30.0%	25.0%	29.6%	33.3	13.9%	12.5–31.2
	Not very easy	7.1%	0	25.0%	0	0	0.7%	6.2

*PCP* patient and carer participation

### Revisions to the learning resource content

Participants suggested the need for clearer examples and summaries. We added ‘information source’ labels and concise ‘summary’ to each case story and simplified language to ensure consistency across all units.

### Development and pilot testing of the full learning resource

#### Cycle 1

##### Participants

Four participants who were not involved in the pilot testing of the prototype unit were invited, and all agreed to attend (100.0%), with three (75.0%) completing the pilot testing (see Table 2).

##### Participants’ feedback

Table 3 and Fig. 4 provide a summary of patient and carer participants’ responses across nine units for each evaluation question:

- **Content alignment with learning outcomes:** Overall, all participants rated the content as ‘well aligned’, with an average of 88.9% rating it as ‘very well aligned’. Specifically, ratings ranged ‘very well aligned’ from 33.3% to 100.0% across units.
- **Ease of understanding:** Overall, all participants found the content ‘easy to understand’, with an average of 77.8% rating it ‘very easy to understand’. Ratings varied from 33.3% to 100.0% across units.
- **Relevance to cancer-impacted individuals:** Overall, all participants deemed the content ‘relevant’ to the needs of cancer-impacted individuals, with an average of 96.3% rating it ‘very relevant’. Ratings ranged from 66.7% to 100.0% across units.
- **Ease of navigation:** Overall, all participants found the units ‘easy to navigate’, with an average of 70.4% rating them ‘very easy to navigate’. Ratings ranged from 66.7% to 100.0% across units.

**Fig. 5:**
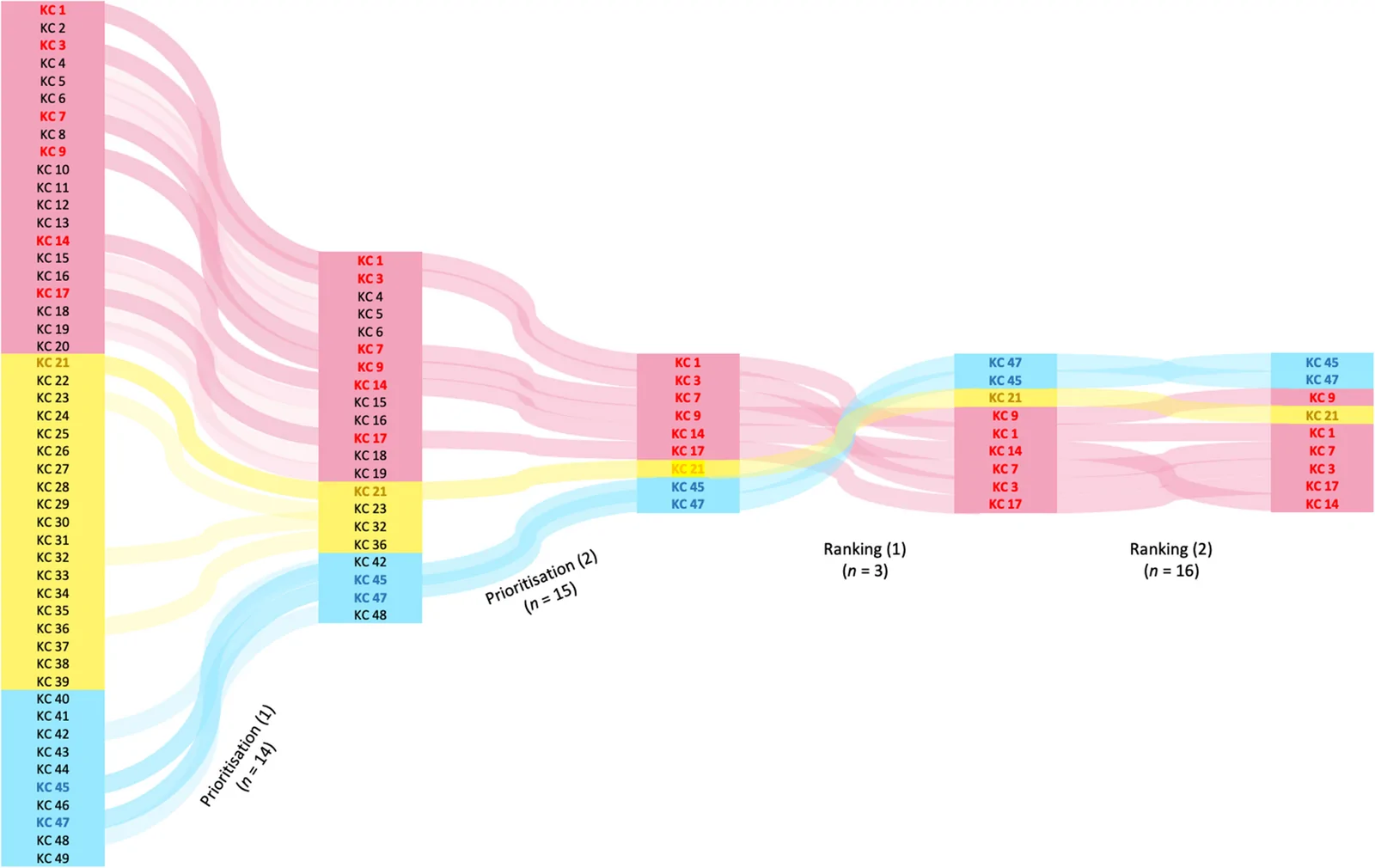
Ranking of the units. The three colours represent the three types of Key Concepts within the Informed Health Choices initiative: pink for concepts about ‘claims’, yellow for ‘comparisons’, and blue for ‘choices’. Only concepts selected at each stage are shown with connecting lines; unselected concepts don not progress. Further details about the Key Concepts prioritisation process are available in an earlier stage of the project [50]

Additionally, the first round of ranking produced an initial order of nine concepts (see Fig. 5). Concepts related to enabling participants to make informed choices and recognise reliable evidence were ranked higher [42], reflecting their perceived relevance to participants.

#### Revisions to the learning resource content

Feedback suggested modifications to quiz clarity, multimedia fluency, and platform navigation. Changes were made and an ‘introduction’ unit for overarching guidance and a ‘conclusion’ unit were added to consolidate key leanings.

*PCP*, patient and carer participation.

#### Cycle 2 pilot testing

##### Participants

A total of 40 participants were invited to participate in this pilot testing. Of these, 12 had previously taken part in the prioritisation stage but were not involved in the review or pilot testing of any earlier units, and 28 were those who expressed interest during the early recruitment stages of the IHC-C programme but had not participated in any previous stage. Additionally, to enhance the completion rate and ensure diversity among participants, a small-scale social media recruitment effort was conducted, resulting in the addition of two new participants.

In total, 23 participants (54.8%) agreed to participate Among these, eight had completed the prioritisation stage [50], seven were early recruits with no prior involvement, and one was newly recruited via social media. Three participants were excluded to maintain a diverse participant pool, as their characteristics overlapped with those of other participants regarding age groups or cancer experience, limiting the opportunity to bring new perspectives to the learning resource. Ultimately, 16 participants (80.0%) completed this cycle of pilot testing (see Table 2).

##### Participants’ feedback — quantitative results

Table 3 and Fig. 4 summarise participants’ responses across nine units for each evaluation question:

- **Content alignment with learning outcomes:** Overall, 98.6% of participants rated the content as ‘well aligned’, with an average of 82.6% rating it ‘very well aligned’. Ratings ranged from 62.5% to 93.8% across units.
- **Ease of understanding:** Overall, all participants found the content ‘easy to understand’, with an average of 80.6% rating it ‘very easy to understand’. Ratings varied from 68.8% to 93.8% across units.
- **Relevance to cancer-impacted individuals:** Overall, all participants deemed the content ‘relevant’ to the needs of cancer-impacted individuals, with an average of 84.7% rating it ‘very relevant’. Ratings ranged from 75.0% to 100.0% across units.
- **Ease of navigation:** Overall, 99.3% of participants found the units ‘easy to navigate’, with an average of 85.4% rated ‘very easy to navigate’. Ratings varied from 68.8% to 100.0% across units.

Additionally, the rankings were refined through further input (see Fig. 5), with the concepts related to informed choices maintaining their positions and others experiencing minor adjustments, representing an evolving consensus among participants.

##### Participants’ feedback — qualitative results

During each pilot testing, qualitative feedback from open-ended questions about participants’ experiences and suggestions for improving the learning resource were collected. However, only the feedback from this cycle of pilot testing was formally analysed, as previous rounds yielded limited input relevant to revising the learning resource content.

Two main themes were developed following the analysis of participant feedback (Table 4). The first theme, ‘Perceived Strengths of the IHC-C Learning Resource’, encompassed effective learning materials design, clarity and content engagement, comprehensive and balanced information delivery, knowledge acquisition and practical application, and critical thinking and decision-making reflection. The second theme, ‘Suggestions for Improvement of the IHC-C Learning Resource’, focused on content improvement, accessibility improvement, and incorporating more resources and additional materials.

**Table 4:** Perceived strengths and suggestions for improvement of the learning resource (*n* = 16)

Theme and subtheme categories	Illustrative quotes
Perceived Strengths of the Informed Health Choices-Cancer Learning Resource (total number of coded quotes, 615)	
Effective learning materials design	‘Very important topics when trying to navigate treatment decisions.’ (Male, above 64, a survivor) ‘…it comes with examples from different backgrounds and choices that people have made… it's very credible as these examples are based on real people with accompanied links so they can be checked out.’ (Female, 35–44, a loved one of a patient) ‘I liked all the examples of research, clips from social media, and the case stores. They gave very different and diverse examples.’ (Female, 55–64, a survivor) ‘In terms of the lay out, graphics, videos & transcripts—all of these were user friendly and appropriate.’ (Female, 45–54, informal caregiver of a patient) ‘…the visuals are excellent…’ (Female, 45–54, current patient)
Clarity and content engagement	‘Reinforcing knowledge in an easy-to-follow way – suitable for people of varying health literacy.’ (Female, above 64, a survivor) ‘All language used is clear and understandable which makes for easy read of programme—forearms patient.’ (Female, 55–64, current patient) ‘The practical, easy to relate to way the programme is delivered.’ (Female, above 64, a survivor) ‘…straight forward… the material is presented in an accessible way…and also provokes wanting to learn more.’ (Female, above 64, survivor)
Comprehensive and balanced information delivery	‘It gave a very balanced view on treatments stating that research combined with conversations…’ (Female, 55–64, a survivor) ‘Overall I fully enjoyed every aspect of the programme… This is the answer to many patients prayers and having all the necessary information under one umbrella is exactly what a patient needs to forearm them and ensure they make the best choice.’ (Female, 55–64, current patient) ‘It was very balanced and very relevant to people dealing with a distressing diagnosis and trying to choose what would work best for them.’ (Female, 55–64, a survivor) ‘This is all relevant information.’ (Female, 35–44, a survivor)
Knowledge acquisition and practical application	‘…it was an eye opener, it shows what is important to you on making the right decisions for you in my opinion.’ (Female, 35–44, a loved one of a cancer patient) ‘It provided food for thought and helpful ways to assist a friend or loved one who may be straying far from proved scientific evidence in their choice and promotion of unproven treatments. I have a family member who is obsessed with… being a cure all for all known medical conditions including cancer.’ (Female, above 64, a survivor) ‘Good realisation… Good reading about… – found these new information very interesting… I learned how to spot misinformation… Lots of great catchy quotes that I will long remember…’ (Female, 55–64, current patient) ‘This is all information I would have loved to have had when I was first diagnosed…’ (Female, 35–44, a survivor)
Critical thinking and decision-making reflection	‘I would definitely approach new treatments with caution having studied this unit… Critical thinking skills are essential when we need to consider new treatments.’ (Female, 45–54, a loved one of a patient) ‘‘Think critically and make better choices'is now one of my favourite quotes.’ (Female, 55–64, a current patient) ‘The skill set needed to think critically is developed in a gradual & logical manner, building up the confidence of the user as they start to identify misinformation trends and apply a check list to articles & claims. I think that this programme is a positive and realistic approach to building these skills for people who are potentially worried, under pressure, unwell & facing huge life changes.’ (Female, 45–54, a loved one of a patient) ‘The learning is genius and if I had not been here before it would most definitely be sending me in the right direction and ensuring I made the best possible informed choices for me.’ (Female, 55–64, current patient)
Suggestions for Improvement of the Informed Health Choices-Cancer Learning Resource (total number of coded quotes, 198)	
Content improvement	‘‘Let’s read some posts… on how’ – suggest using review or look at instead of ‘read’ as different media types are involved.’ (Female, 45–54, a current patient) ‘Characteristic 2 – the effects of a new or expensive treatment are claimed larger than they are – this sentence segment would be more readable if worded slightly differently.’ (Female, 45–54, a current patient) ‘It is very clear & uses appropriate language, perhaps some of the language is tending towards more academic terminology in some of the units describing narratives & decision making, but in general it is easily understood.’ (Female, 45–54, informal caregiver of a patient) ‘The true or false question got me in the Quiz. I think the wording is not clear enough. I know I should have marked false but marked true.’ (Female, 35–44, an informal caregiver of a patient)
Accessibility improvement	‘The'Mark as Done'button may be better placed at the end of each content section, rather than at the beginning.’ (Male, 25–34, an informal caregiver of a patient) ‘…font very small in some of the social media posts and was a little blurry when zoomed up.’ (Female, 45–54, a loved one of a patient) ‘The display/navigation was a little confusing at first. For general roll out I think it would be beneficial to look at options to make it more intuitive.’ (Female, 45–54, a current patient) ‘The introduction video froze a few times but this may have been the connectivity. No problems with the other videos though.’ (Female, 35–44, an informal caregiver of a patient) ‘The speed of speech in the video explaining… was too fast. The graphics in the video were helpful. The script describing clinical trials was helpful also. For a visually impaired person the audio in the video would be very important, so the speed of speech needs to be clearer.’ (Female, 55–64, a survivor) ‘Also would there be an option for an audio of the written sections? This would help in terms of accessibility.’ (Female, 45–54, a loved one of a patient)
Incorporating more resources and additional materials	‘Perhaps another video or interview with a person with cancer who explains… for treatment choices made.’ (Female, above 64, survivor) ‘Perhaps an A to Z link to reputable sites…could be included in programme, as I think this would be a deterrent to visit sites further afield.’ (Female, 55–64, current patient) ‘Creating a practical checklist and/or tools could be very useful for patients, especially those with lower educational levels. Also, it would be really great to have this course in other language…’ (Male, 25–34, an informal caregiver of a patient) ‘I would add a unit on genetic testing. This is a big reality for many cancer patients and can present both opportunities and dilemmas…’ (Female, 55–64, a survivor) ‘Suggest a toolkit section at the end of each lesson with printable pdf of the lesson, useful templates, questions to use, a how to execute the lessons in practical steps, and an overall wallet sized insert with a quick reference guide covering all lessons…’ (Female, 45–54, a current patient)

Participants identified several aspects of the learning resource that facilitated their learning outcomes effectively. The content structure, presentation design, and diverse material formats were highlighted as key strengths. The information was regarded as relevant to real-life scenarios, while the language was described as clear, engaging, and comprehensive. The content delivery was perceived as balanced, offering multiple perspectives. Participants reported gaining new knowledge and strategies and intended to apply them in real-life contexts. The learning resource was also praised for fostering critical thinking, particularly in evaluating information and making informed decisions about health.

Participants provided feedback on areas for enhancement, focusing on clarity, accessibility, and resource offerings. Suggestions included refining language for improved readability, enhancing interface usability, and incorporating additional practical tools and resources to address diverse learning needs. These recommendations aim to create a more user-friendly and adaptable learning resource, catering to the varying profiles and preferences of its users.

#### Technical support and participant queries

During the review period, two participants reached out via email for assistance. One sought clarification about navigating between units on the learning platform, and the other had difficulty accessing the evaluation form. Both issues were resolved within 24 h.

#### Revisions to the learning resource content

Refinements focused on standardised quiz complexity, enhancing multimedia materials, and aligning terminology. Suggestions regarding resource expansion were considered beyond the scope of the current development. Additional platform adjustments, including refining the layout and visual flow of content pages, improving navigation features (e.g. clearer progress indicators and return buttons), and simplifying buttons, on-screen instructions, and headings, were implemented to address final concerns related to clarity.

### Final learning resource

**Fig. 6:**
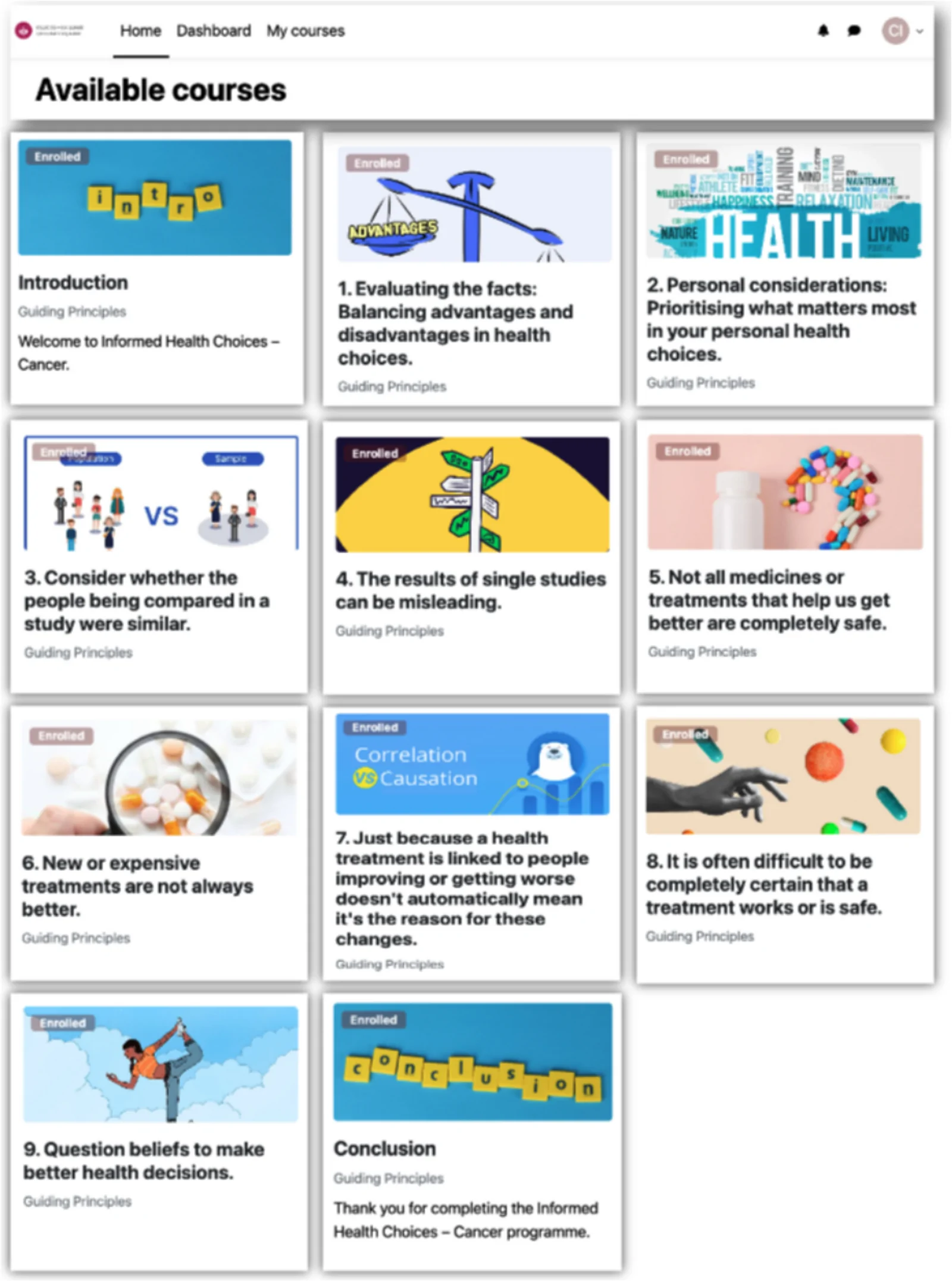
IHC-C learning resource on online platform

The final learning resource includes nine standalone units, along with an introduction unit and a conclusion unit (see Fig. 6). All revisions based on participant feedback were incorporated to enhance clarity, consistency, and accessibility. The resource is designed to foster critical thinking, support informed decision-making, and address the diverse needs of individuals impacted by cancer.

## Discussion

This study developed an online learning resource using human-centred design with iterative refinement. The pilot testing demonstrated the critical value of diverse stakeholder involvement, particularly those directly impacted by cancer. This was evidenced by the marked difference in relevance ratings between the patient and carer group (100%) and project group (50%) during the prototype phase. These findings align with previous research on the importance of end-user involvement in educational intervention development [55, 56].

The patient and carer group’s diversity in cancer diagnoses, stages, and timelines enhanced the resource’s accessibility, though these factors did not significantly influence health literacy [54]. While older participants tended to provide more positive feedback, possibly reflecting age-related differences in evaluation approaches [54], their responses reinforced the need for reliable, accessible resources to support informed decision-making.

The development of the learning resource was informed by approaches from other Informed Health Choices programmes [38, 44, 47, 57] while integrating iterative refinement cycles to address challenges and enhance quality [58, 59]. For example, in our research, navigation ratings (‘very easy to navigate’) showed progressive improvement across development phases: prototype (50.0%), first pilot cycle (70.4%), and final cycle (85.4%), demonstrating the value of iterative refinement. Such iterative processes not only improve usability but also reinforce the value of user-centred development for online educational tools, suggesting that future development of such educational tools can take the iterative process as an effective method in development.

Through iterative cycles integrating patient and carer input, we systematically refined the learning resource’s content, language, and multimedia elements. Early feedback prompted the addition of source labels and summaries; later insights led to improved quiz clarity, platform navigation, and alignment of complexity across units. This process illustrates how sustained stakeholder engagement can meaningfully shape educational tools, aligning them more closely with user needs. Our findings parallel research on user-centred design, suggesting that increasing evidence-based refinements boost accessibility and relevance, which are also key factors in fostering informed decision-making and engagement [52, 57].

The development of the learning resource content integrated, evidence-based information from cancer care frameworks with contextualised Key Concepts from prior IHC initiatives [38, 43, 44, 47]. The resource featured multimedia formats, including real-life examples, case stories, and practical tips grounded in local experiences; this approach reflected best practices in designing educational resources targeting diverse populations [60–62]. Pilot testing results showed strong content alignment with learning outcomes, with endorsement ratings of the alignment as ‘very well’ from 71.4% for the prototype unit to 82.6% for the final iteration of the full learning resource. Qualitative feedback further highlighted the effectiveness of prioritising relatable real-life scenarios before presenting instructional content. This structure not only enhanced engagement but also reinforced participants’ ability to apply the learning to their own contexts.

Plain language was a central principle of the resource’s development to ensure accessibility and ease of understanding across diverse literacy levels. For example, we avoided technical medical terminology and used everyday language to explain complex concepts. As tailoring language to diverse educational needs is essential yet challenging [63, 64], stakeholders played an important role in reviewing and refining the language during each development cycle to ensure clarity and simplicity. This collaborative effort resulted in progressive improvements in ease-of-understanding ratings, rising from 71.4% rated the learning resource as ‘very easy’ to understand in the prototype stage to 80.6% in the final pilot. Nevertheless, qualitative feedback still identified areas for further improvement, particularly in tailoring the resource to linguistic and cultural nuances. These findings highlight the ongoing challenges in creating universally accessible educational materials and emphasise the necessity of sustained stakeholder engagement and iterative refinement.

A strength of this study lies in its integration of diverse stakeholder perspectives through iterative development cycles, ensuring the resource effectively meets the practical needs of its intended audience. The iterative refinement process enhanced navigation, usability, and engagement, highlighting the benefits of a human-centred approach. These findings suggest that future educational initiatives targeting individuals impacted by health conditions should prioritise relatability, strategic content organisation, and accessibility. Iterative feedback mechanisms and active stakeholder involvement not only enhance the effectiveness of the learning resource development but also ensure alignment with the diverse needs and expectations of target populations. By prioritising these principles, educational resources can foster meaningful learning experiences and empower individuals to achieve intended outcomes.

Two key limitations affect the generalisability of our findings. First, the gender imbalance in our sample (predominantly female participants) limits our understanding of how the resource might serve different gender groups [31]. Second, the overrepresentation of participants with advanced educational backgrounds [50] raises uncertainty about the resource’s effectiveness for users of varying educational levels. Future research should address both limitations through targeted recruitment strategies. As education level significantly impacts health literacy [31], future initiatives should adopt targeted recruitment strategies to engage participants across a broad range of educational backgrounds. In addition, future learning resources should incorporate culturally tailored approaches and involve stakeholders from diverse backgrounds to further enhance inclusivity.

## Conclusion

The proliferation of cancer-related information and misinformation creates critical challenges for those impacted by cancer. Through the Informed Health Choices-Cancer programme, we developed and validated an online learning resource using human-centred design and iterative refinement. The involvement of diverse stakeholders, including individuals impacted by cancer, healthcare professionals, and researchers, ensured that the resource was practical, relevant, and accessible. Feedback from pilot testing demonstrated that the resource was well-received, with participants rating it as comprehensive, engaging, and tailored to varying literacy levels. The iterative design process enabled continuous improvement, particularly in addressing usability challenges and refining content to meet the real-life needs of cancer-impacted individuals.

The findings underscore the value of involving end-users throughout the development process, ensuring that educational resources align with the lived experiences and preferences of the target audience. The Informed Health Choices-Cancer programme offers an innovative approach for integrating critical thinking into cancer care through a user-centred, evidence-based educational resource. Preliminary findings suggest it has the potential to empower individuals to make informed decisions about their health while possibly supporting healthcare professionals in their educational roles. However, further evaluation is needed to establish its effectiveness across diverse contexts. A randomised controlled trial is now needed to evaluate the resource’s effectiveness in improving critical health decision-making skills among cancer-impacted individuals.

## Data Availability

No datasets were generated or analysed during the current study.
